# Effect of Antibiotics on the Microbial Efficiency of Anaerobic Digestion of Wastewater: A Review

**DOI:** 10.3389/fmicb.2020.611613

**Published:** 2021-01-28

**Authors:** Leilei Xiao, Yiping Wang, Eric Lichtfouse, Zhenkai Li, P. Senthil Kumar, Jian Liu, Dawei Feng, Qingli Yang, Fanghua Liu

**Affiliations:** ^1^Key Laboratory of Coastal Biology and Biological Resources Utilization, Yantai Institute of Coastal Zone Research, Chinese Academy of Sciences, Yantai, China; ^2^Key Laboratory of Coastal Environmental Processes and Ecological Remediation, Yantai Institute of Coastal Zone Research (YIC), Chinese Academy of Sciences (CAS), Yantai, China; ^3^Department of Chemical Engineering, Sri Sivasubramaniya Nadar College of Engineering, Chennai, India; ^4^Aix-Marseille Univ, CNRS, IRD, INRAE, Coll France, CEREGE, Aix en Provence, France; ^5^State Key Laboratory of Multiphase Flow in Power Engineering, Xi’an Jiaotong University, Xi’an, China; ^6^School of Resources and Environmental Engineering, Ludong University, Yantai, China; ^7^College of Food Science and Engineering, Qingdao Agricultural University, Qingdao, China; ^8^Shandong Key Laboratory of Biophysics, Shandong Engineering Laboratory of Swine Health Big Data and Intelligent Monitoring, Institute of Biophysics, Dezhou University, Dezhou, China

**Keywords:** antibiotics, anaerobic digestion, biomethane, fermentative bacteria, methanogenic archaea

## Abstract

Recycling waste into new materials and energy is becoming a major challenge in the context of the future circular economy, calling for advanced methods of waste treatment. For instance, microbially-mediated anaerobic digestion is widely used for conversion of sewage sludge into biomethane, fertilizers and other products, yet the efficiency of microbial digestion is limited by the occurrence of antibiotics in sludges, originating from drug consumption for human and animal health. Here we present antibiotic levels in Chinese wastewater, then we review the effects of antibiotics on hydrolysis, acidogenesis and methanogenesis, with focus on macrolides, tetracyclines, β-lactams and antibiotic mixtures. We detail effects of antibiotics on fermentative bacteria and methanogenic archaea. Most results display adverse effects of antibiotics on anaerobic digestion, yet some antibiotics promote hydrolysis, acidogenesis and methanogenesis.

## Introduction

Antibiotics are widely used to treat human and animal diseases, yet the overuse of antibiotics is inducing issues of pollution, development of antibiotic resistance by pathogenic bacteria, and inhibition of engineered microbial processes such as anaerobic digestion of wastewater. The global consumption of antimicrobials in livestock production was estimated at more than 63 thousand tons in 2010, and is projected to rise to 105 thousand tons by 2030 (Yadav and Kumar, 2020). In the 2010s, countries with the largest share of global antimicrobial consumption in livestock production were China (23%, in weight), United States of America (USA, 13%), Brazil (9%), India (3%), and Germany (3%). Projections show that by 2030 the main antibiotic-consuming nations should be China (30%), United States (10%), Brazil (8%), India (4%), and Mexico (2%) (Van Boeckel et al., 2015). Other reports estimate the annual consumption of antibiotics in China at about 150,000 tons to 200,000 tons in recent years, which is nearly ten times that of the United States and 150 times that of the United Kingdom (Larson, 2015; Zhang et al., 2015; Yadav and Kumar, 2020). The extensive use of antibiotics leads to their continuous enrichment through wastewater and sewage and eventual exposure to the natural environment (Chen et al., 2016; Liu et al., 2018; Qiao et al., 2018; Alonso et al., 2019). The strong adsorption capacity of sludge toward antibiotics enhances the accumulation of antibiotics in sludge. Therefore, antibiotic residues and their effects in sludge have attracted major attention (Ju et al., 2016; Shin et al., 2020; Yu et al., 2020; Xiao et al., 2021).

Anaerobic digestion involves processes during which microorganisms breakdown biodegradable organic material in the absence of oxygen (Chen et al., 2008). Digestion starts with bacterial hydrolysis of organic polymers such as proteins that are broken down into amino acids that feed bacteria. Sugars and amino acids are then converted by acidogenic bacteria into carbon dioxide, hydrogen, ammonia and organic acids. Acetogenetic bacteria convert these organic acids into acetate, and additional ammonia, hydrogen and carbon dioxide. Finally, these products are converted into methane and carbon dioxide by methanogens. Anaerobic digestion is widely used in the treatment of sewage sludges to reduce sludges volume and to produce methane (Mehariya et al., 2018; Xiao et al., 2019b).

The efficiency of anaerobic digestion is reduced by the presence of antibiotics and antibiotic residues, which inhibit the microbial community and activity, thus further limiting the efficiency of the entire anaerobic digestion system (Kovalakova et al., 2020; Rusanowska et al., 2020). Accordingly, existing pre-treatments are aimed to reduce levels of antibiotic residues (Gurmessa et al., 2020). For example, Pei et al. (2015) studied the effect of ultrasonic and ozone pre-treatments on pharmaceutical waste activated sludge’s solubilization and anaerobic biodegradability. Thermal hydrolysis pretreatment shows removal of fluoroquinones in sewage sludge during anaerobic digestion (Li et al., 2017). Research on the effects of antibiotics on anaerobic digestion has rapidly grown in the last decade (Walsh, 2000; Kohanski et al., 2007), yet comprehensive reviews are still rare (Cheng et al., 2018; Yang et al., 2019). Therefore, this paper takes China as an example to systematically summarize the general usage of antibiotics at present and reviews the main effects of antibiotics on anaerobic digestion with anaerobic biomethane production as the critical point.

## Antibiotic Utilization Taking China as an Example

At present, antibiotics are mainly used in hospitals and livestock farms (Sim et al., 2011). Residual antibiotics are then transferred into wastewater where they are partly degraded and partly preserved. For instance, Table 1 shows that antibiotic concentrations range from 0.046 to 4.552 μg/L in medical wastewater and from 0 to 130.67 μg/L in livestock wastewater. Tetracyclines, β-lactams, sulfonamides and quinolones are found in medical and livestock wastewater (Wei et al., 2011; Zhang and Li, 2018). Medical wastewater also contains macrolides, which are mostly used in human clinical treatments (Wei et al., 2011; Li et al., 2013). However, compared with medical wastewater, animal wastewater contains higher levels of tetracyclines and sulfonamides, which are used to prevent and treat livestock diseases (Zhang and Li, 2018; Sun et al., 2019). The antibiotic contents in wastewater are variable in different regions (Table 1), such as 1.41 μg/L in Henan and less than 0.38 μg/L in Xinjiang for tetracycline. Wei et al. (2011) found that the antibiotic contents in wastewater are different within Jiangsu province, which is thought to be caused by the diverse utilization of antibiotics in different cities. The excessive use of antibiotics has received sufficient attention from the government and scientific researchers in China. As a consequence, a series of policies have been introduced, in aquaculture for instance (Broughton and Walker, 2010).

**TABLE 1:** Antibiotic levels in medical and livestock wastewater in some regions of China.

**Type**	**Name**	**Concentration (μg/L)**	**Wastewater source**	**Province/city**	**References**
Macrolides	Erythromycin	0.48	Medical	Henan	Wei et al., 2011
Tetracyclines	Tetracycline	1.41	Medical	Henan	Wei et al., 2011
		0.046–0.374	Medical	Xinjiang	Li et al., 2013
		31.05	Livestock	Shanghai	Sun et al., 2019
		3.01–8.58	Livestock	Tianjin	Lu et al., 2014
	Terramycin	1.48	Medical	Henan	Wei et al., 2011
		0.042–0.448	Medical	Xinjiang	Li et al., 2013
		60.50	Livestock	Shanghai	Sun et al., 2019
		60.15–82.59	Livestock	Tianjin	Lu et al., 2014
	Aureomycin	0.55–130.67	Livestock	Tianjin	Lu et al., 2014
β-lactams	Cefalexin	2.39	Medical	Henan	Wei et al., 2011
	Amoxicillin	4.99	Livestock	Tianjin	Lu et al., 2014
Sulfonamides	Sulfamethoxazole	0.53	Medical	Henan	Wei et al., 2011
		0–37	Livestock	Jiangsu	Zhang and Li, 2018
	Sulfadimidine	0–70	Livestock	Jiangsu	Zhang and Li, 2018
	Sulfadiazine	0.086–0.574	Medical	Xinjiang	Li et al., 2013
		0–2.248	Medical	Hainan	Wang et al., 2018
		0.21–0.68	Livestock	Tianjin	Zhang and Li, 2018
		0–62	Livestock	Jiangsu	Zhang and Li, 2018
Quinolones	Ciprofloxacin	1.33	Medical	Henan	Wei et al., 2011
		0.592–4.552	Medical	Hainan	Wang et al., 2018
		13.56	Livestock	Tianjin	Lu et al., 2014
	Norfloxacin	1.63	Medical	Henan	Wei et al., 2011
	Ofloxacin	2.33	Medical	Henan	Wei et al., 2011
		0.377–0.942	Medical	Xinjiang	Li et al., 2013

## Effects of Antibiotics on Anaerobic Digestion

Microbes transform complex organic substances into methane through anaerobic digestion, which is the primary way of anaerobic mineralization of organics. The process of biomethane production from macromolecular organic carbon involves a variety of microbes, mainly fermenting bacteria and methanogenic archaea. The accumulation of antibiotics dissolved in wastewater into sludge can affect the microbial community associated with each stage of anaerobic digestion (Cheng et al., 2018; Zhi and Zhang, 2019; Luo et al., 2020). Specially, the influencing mechanisms of different antibiotics are various (Walsh, 2000; Kohanski et al., 2007). It is discussed in detail in the following parts.

### Effects of Antibiotics on the Hydrolytic and Acidogenic Stages of Anaerobic Digestion

The hydrolysis stage is considered as the main rate-limiting step of anaerobic digestion (Mata-Alvarez et al., 2000; Carlsson et al., 2012; Gonzalez et al., 2018). During hydrolysis, partly insoluble sludge macromolecules such as proteins, carbohydrates, and lipids are converted into more soluble, smaller molecular substances. Then, during the acidification stage, the hydrolysates are further converted into volatile fatty acids (VFAs), including acetate, propionate, and butyrate.

Table 2 summarizes the effects of antibiotics on the successive steps of anaerobic digestion. Concerning hydrolysis and acidification, data shows the inhibition of the propionate metabolism by two antibiotics, of the butyrate metabolism by five antibiotics and of organic degradation by one antibiotic. Here, inhibiting antibiotics are macrolides and tetracyclines. For **β**-lactams, an absence of effect on organic degradation has been shown for cefalexin.

**TABLE 2:** Effects of antibiotics on the diverse stages of anaerobic digestion and biomethane production.

**Type**	**Name**	**Hydrolysis and acidification**			**Methane production**			**References**
		**Propionate metabolism**	**Butyrate metabolism**	**Organic degradation**	**Acetoclastic**	**Hydrogenotrophic**	**Methylotrophic**	
Macrolides	Roxithromycin	No data	No data	Inhibition	Inhibition	No data	No data	Ni et al., 2020
	Erythromycin	No data	Inhibition	No data	Inhibition	No data	No effect	Zhang and Li, 2018
	Tylosin	Inhibition	Inhibition	No data	No effect	No data	No data	Sanz et al., 1996
Tetracyclines	Terramycin	Inhibition	Inhibition at high concentration	No data	Inhibition	No data	No data	Tian et al., 2018
	Tetracycline	No data	Inhibition	No data	Inhibition	No data	No data	Zhang and Li, 2018
	Aureomycin	No data	Inhibition	No data	Inhibition	No data	No data	Sanz et al., 1996
β-lactams	Cefalexin	No data	No data	No effect	Inhibition at low concentration Enhancement at high concentration	No data	No data	Lu et al., 2014

#### Effects of Antibiotics on the Hydrolytic Stage

Erythromycin’s long-term action inhibits microbial growth, reduces hydrolysis rate, blocks substrate storage, and accelerates endogenous respiration (Pala-Ozkok and Orhon, 2013). In contrast, the presence of azithromycin analogs in sludge leads to high methane production because of the increased hydrolysis efficiency that induces an increased proportion of fermentative bacteria and archae at the methanogenesis stage (Mustapha et al., 2018). Clarithromycin enhanced the production of VFAs during hydrolysis and acidogenesis of activated sludge, as a possible result of disruption and solubilization of extracellular polymeric substances (Huang et al., 2019). Yet clarithromycin inhibited acid consumption and, in turn, inhibited strongly hydrolysis and acidogenesis. In addition, species responsible for hydrolysis and acidogenesis were slightly more abundant with clarithromycin, whereas the abundance of acid-consuming microorganisms such as *Gamma-proteobacteria* and *Rhodobacter* declined. Overall, some macrolides and tetracyclines inhibit clearly hydrolysis and acidification, and more investigations are needed to confirm other isolated findings.

#### Effects of Antibiotics on the Acidogenic Stage

A study showed that roxithromycin macrolide and sulfamethoxazole increased the production of VFAs in anaerobic fermentation of waste activated sludge, which is explained by affecting microbial diversity and disruption of extracellular polymeric substances (Chen et al., 2020; Miritana et al., 2020). On the contrary, Ni et al. (2020) found that roxithromycin inhibits acidogenesis and methanogenesis in anaerobic digestion of activated sludge, leading to a decreased methane production. They also found that exposure to roxithromycin increased the abundance of antibiotic resistance genes (ARGs), esterases, methylases and phosphorylases in the digested sludge. Carneiro et al. (2020) suggest that the co-metabolic transformation of organic antibiotics is mainly achieved during acidogenesis. The influence of antibiotics on the acidogenic stage may also be closely related to the diversity, abundance, and activity of microorganisms.

The antibiotic concentration is a factor controlling effects on acidogenesis. For example, a high concentration of tylosin inhibited propionate and butyrate metabolisms during the anaerobic fermentation process of pig manure (Table 2; Stone et al., 2009). By contrast, a low tylosin concentration had no significant effect on the overall system performance, and, in particular, there was no change in the utilization of propionate, butyrate and acetate. In addition, some other studies presented similar trends (Cetecioglu et al., 2012; Tian et al., 2018). The mechanisms of inhibition may be explained by the accumulation of acidic intermediates that decrease the pH of the whole system. It was proposed that tylosin has negative effects on methanogenesis through its inhibition of propionate- and butyrate-oxidizing syntrophic bacteria and fermenting bacteria (Stone et al., 2009). Overall, there is convergent evidence that some macrolides and tetracyclines inhibit acidogenesis. Therefore, pretreatments should be performed to reduce antibiotic contents in order to improve further fermentation (Alamo et al., 2020; Gurmessa et al., 2020; Tao et al., 2020).

#### Effects of Antibiotics on Fermentative Bacteria

Bacteria having hydrolysis and acidification functions play an important role in the early stage of anaerobic digestion, in which these microbes convert larger molecules of organic matter in sludge into simple smaller molecules and then into VFAs, and consequently affect the overall efficiency of anaerobic digestion. In the last ten years, researchers focused on interaction between antibiotics and fermentative microbes. Some mechanisms of antibiotics affecting anaerobic fermentation have been elucidated. For example, macrolides and tetracyclines are inhibitors of protein synthesis in bacteria (Qiao et al., 2018), inhibiting bacteria’s normal growth. This may be the reason why these two types of antibiotics inhibit the bacteria having hydrolysis and acidification functions. The other mechanisms are concluded differently. Macrolides and tetracyclines bind to the 23S rRNA of the ribosomal large subunit and the 16S rRNA of the ribosomal small subunit, respectively (Vester and Douthwaite, 2001), which affect the function of ribosomes.

Different antibiotics of the same type also have diverse effects on fermentative bacteria. For example, both erythromycin and tylosin belonging to macrolides inhibit butyrate producing bacteria to a certain extent, leading to the accumulation of VFAs and the instability of the whole system (Amin et al., 2006). However, a recent study revealed that roxithromycin exposure affected the waste activated sludge anaerobic digestion and the change of ARGs in the anaerobic digestion, accompanied by the inhibition of acidogenesis and methanogenesis, leading to decreased methane production (Ni et al., 2020). Similarly, long-term exposure to tylosin directly inhibits the propionate-oxidizing syntrophic bacteria which closely relate to *Syntrophobacter*, thus indirectly inhibited *Methanosaeta* by high propionate concentrations and low pH, resulting in the long-term reactor failure (Shimada et al., 2011).

Compared to macrolides, fewer studies were conducted about the effects of tetracyclines and β-lactam on anaerobic fermentation. Though terramycin and aureomycin are both tetracyclines, they have different effects on anaerobic fermenting bacteria. Specially, the mechanisms ruling the effects of different concentrations of antibiotics on fermenting bacteria are also distinct. At a low concentration (50 mg/L), oxyterramycin significantly inhibited propionate oxidizing bacteria, and shown significantly lower degradation rate of propionate than that of the control group without antibiotics (Arikan et al., 2006). However, the metabolic activity of butyrate oxidizing bacteria was little affected, so that the butyrate content in VFAs was extremely low. At a high concentration (500 mg/L), terramycin can further enhance the inhibition of propionate oxidizing bacteria and, at the same time, inhibit butyrate oxidizing bacteria (Arikan et al., 2006).

β-lactam inhibitors can inhibit the synthesis of cell wall murein by inhibiting penicillin-binding protein, which can cause bacterial cell wall defects (Sanz et al., 1996). β-lactams can stimulate the synthesis of autolytic enzymes, thus accelerating bacterial death. Cefalexin belonging to β-lactams can stimulate the secretion of exopolysaccharides, which act as a protective layer of microbial cells and create a suitable environment for microbial growth and methanogenesis. Studies have shown that bacteria in urban sludge currently have higher drug resistance to β-lactams and lower resistance to tetracyclines. For example, the antibiotic resistance to ampicillin, cefalotin, and cefotaxime could be elevated through anaerobic digestion (LaPara et al., 2011).

### Effects of Antibiotics on the Methanogenic Stage of Anaerobic Digestion

The methanogenic stage, as the final step of anaerobic digestion, is the key step of substrate fermentation. Three processes are actually known to contribute to methane production: CO_2_ reduction, acetate dismutation, and methylotrophic methanogenesis. First, about the two-thirds of biomethane production is explained by acetoclastic methanogenesis in natural environments. However, in H_2_ upgrading/ammonia inhibited AD system, methane production is mainly from hydrogenotrophic methanogenesis pathway. For acetoclastic methanogenesis, methanogens oxidize carboxyl groups to CO_2_ and generate reducing forces to reduce methyl carbon to methane (Welte and Deppenmeier, 2014; Xiao et al., 2019b, 2020a). At present, only *Methanosarcina* and *Methanosaeta* have been found to produce methane by acetoclastic methanogenesis (Holmes and Smith, 2016; Li et al., 2018; Xiao et al., 2019a).

The second pathway for biomethane production is CO_2_ reduction using hydrogen/formic acid as indirect electron carriers. With the discovery of methane production through direct electron reduction, the theory of “electromethanogenesis” has been gradually recognized and named as direct interspecies electron transfer (DIET) (Morita et al., 2011; Xiao et al., 2018). Overall, direct electron transfer by conductive pili/protein and indirect electron transfer by H_2_/formate work together for CO_2_ reduction to generate biomethane. Third, methylotrophic methanogenesis reduces the methyl group of, e.g., methanol and methylamine, to methane (Yuan et al., 2019; Conrad, 2020); this process contributes only a small amount of methane production in bioengineering. Mustapha et al. (2016) showed the simultaneous functions and interactions of diverse bacteria and methanogenic archaea at different stages of the anaerobic digestion of waste-activated sludge. They found that the proportion of *Caldilinea*, *Methanosarcina*, and *Clostridium* is related to methane production trends after the exposure of azithromycin, chloramphenicol, and kanamycin. The effects on antibiotics are detailed in the next subsections and Table 3.

**TABLE 3:** Effects of different antibiotics on the methane production of anaerobic digestion.

**Type**	**Name**	**Conc. (mg/L)**	**Time (days)**	**T (°C)**	**Methanogenesis**	**References**
Macrolides	Roxithromycin	1	3	35 ± 1	Total methane production declined by ∼15.7%	Ni et al., 2020
		1	30	35 ± 1	Total methane production declined by ∼ 10%	Ni et al., 2020
	Erythromycin	25	7	55	Cumulative methane production decreased by 40.60%∼44.91%	Zhang and Li, 2018
		25	6	35 ± 2	Methane production declined by ∼ 15.8%	Cetecioglu et al., 2012
		250	6	35 ± 2	Methane production declined by ∼ 68.4%	Cetecioglu et al., 2012
		500	6	35 ± 2	Almost no methane produced	Cetecioglu et al., 2012
	Tylosin	25	28	30 ± 2	Inhibiting methane production by 35%	Sanz et al., 1996
		250	28	30 ± 2	Inhibiting methane production by 45%	Sanz et al., 1996
Tetracyclines	Terramycin	40	50	35	No significant change in methane production	Tian et al., 2018
		200	40	35	No significant change in methane production	Tian et al., 2018
		1000	99	35	A 60% reduction in methane production	Tian et al., 2018
		5	∼ 7	35	No change in cumulative methane production	Arikan et al., 2006
		50	∼ 7	35	Methane production reduced by 23.75%	Arikan et al., 2006
		500	∼ 7	35	Methane production reduced by 90.67%	Arikan et al., 2006
		25	7	55	No acute inhibition of thermophilic anaerobic digestion	Zhang and Li, 2018
		50	7	55	No acute inhibition of thermophilic anaerobic digestion	Zhang and Li, 2018
	Tetracycline	25	7	55	No change in cumulative methane production	Zhang and Li, 2018
		50	7	55	Methane production decreased by 36.13%	Zhang and Li, 2018
		25	6	35 ± 2	No significant difference	Cetecioglu et al., 2012
		50	6	35 ± 2	Methane production declined by ∼ 21.1%	Cetecioglu et al., 2012
		500	6	35 ± 2	Almost no gas produced	Cetecioglu et al., 2012
	Aureomycin	150	28	30 ± 2	Methane production reduced to 20%	Sanz et al., 1996
Cephalosporins	Cefalexin	50	25	35 ± 1	Methane production almost completely suppressed	Lu et al., 2014
		200	25	35 ± 1	Methane production almost completely suppressed	Lu et al., 2014
		600	25	35 ± 1	No effect on methane production	Lu et al., 2014
		1000	25	35 ± 1	No effect on methane production	Lu et al., 2014
		2000	25	35 ± 1	Methane production almost completely suppressed	Lu et al., 2014
		200	157	35 ± 1	Methane production increased by 5.7%	Lu et al., 2014
		600	157	35 ± 1	Methane production increased by 30.3%	Lu et al., 2014
		1000	157	35 ± 1	Methane production increased by 63.8%	Lu et al., 2014
		2000	157	35 ± 1	Methane production declined by 12%	Lu et al., 2014
	Cefazolin	25	7	55	Methane production decreased by 43.03–47.49%	Zhang and Li, 2018

#### Macrolides

Macrolides mainly include roxithromycin, erythromycin and tylosin. Cetecioglu et al. (2012) showed that increasing erythromycin concentrations decreased methane production, possibly caused by reduced acetate utilization. Another study of metabolic transformations of VFAs showed that inhibition depended on the concentration of erythromycin (Cetecioglu et al., 2015). The inhibitory impact was variable with the initial erythromycin dose: at lower doses, the VFA mixture was removed entirely but partially utilized, leading to reduced biogas and methane generation, suggesting the analogy of uncompetitive inhibition.

Chen et al. (2020) showed that roxithromycin inhibits more methanogens than hydrolytic bacteria, thus resulting in an accumulation of VFAs. Exposure of roxithromycin was found to reduce the abundance of methanogenic archaea such as *Methanoseata*, *Methanofastidiosum*, and *Methanolinea* (Ni et al., 2020). Some studies demonstrated that methane production could be restored by prolonged duration of antibiotic action, e.g., using roxithromycin and erythromycin (Table 3). Tylosin has a high inhibitory effect on methane production after long-term action, and this inhibitory effect rises with tylosin concentration. Adaptation may also occur. Indeed, a low concentration of tylosin, 0.01 mg/L was enough to inhibit methane production for biomass that has not been previously in contact with tylosin; whereas no inhibition of methanogenesis was observed in digesters acclimated with 0.01–0.065 mg/L of tylosin (Garcia-Sanchez et al., 2016). Overall, macrolides decrease methane production by about 10–100% depending on the antibiotic concentration (Table 3).

#### Tetracyclines

Compared to macrolides, terramycin, tetracycline, and aureomycin belonging to tetracyclines showed similar tendency to suppress methane production. Terramycin, tetracycline and aureomycin tetracyclines do not inhibit methane production below 25 mg/L but inhibit methane production above 500 mg/L (Arikan et al., 2006; Tian et al., 2018). The inhibition of methane production by terramycin decreases with longer exposure time, even at higher concentrations, which is explained by the development of antibiotic resistance by the microbial community.

Temperature is controlling the impact of tetracyclines. For instance, terramycin reduced the cumulative methane production by 23.75% at 35°C (Arikan et al., 2006), while at 55°C, the same concentration of terramycin did not inhibit anaerobic digestion (Alonso et al., 2019). On the contrary, the inhibition of anaerobic digestion by tetracycline increases with temperature (Diehl and LaPara, 2010; Yi et al., 2016). Here, it is speculated that terramycin is likely to be hydrolyzed at higher temperature, thus in turn losing its inhibitory capacity. By compararison, tetracycline displays better thermal stability and is relatively more difficult to be hydrolyzed (Yi et al., 2016).

The type of tetracycline is also controlling the accumulation of VFAs. For instance, tetracycline induces acetate and butyrate accumulation during thermophilic anaerobic digestion, whereas terramycin does not (Alonso et al., 2019). This is important because many studies reveal that the accumulation of acids inhibit methanogenesis (Lee et al., 2017). Herein, VFAs as non-antibiotic factors may also lead to reduced methane accumulation in the presence of tetracycline. A possible reason for accumulated VFAs may be that methanogens are more vulnerable to tetracycline than acetogens and other bacteria. Thus inhibited methanogens may explain the under-performing AD process. Overall, tetracyclines decrease methane production by 0–90% depending on concentration, temperature and compound type (Table 3), and the development of antibiotic resistance previously observed for macrolides is confirmed.

#### β-Lactams

Several investigation show that β-lactams display inhibitory effects on anaerobic digestion (Guerra et al., 2014; Huang et al., 2018). Lu et al. (2014) showed that a lower concentration of cefalexin inhibits methane production during anaerobic digestion of activated sludge, whereas higher concentration had no significant effect on methane production. This behavior was explained by the fact that cefalexin addition induces the excretion of extracellular polymeric substances that form microbial protecting layers, thus providing a suitable environment for microbes’ growth and fermentation. Moreover, the long-term observation of the impact of cefalexin on organic substrate degradation and microbial community structure in a granular sludge bed system showed that the presence of cefalexin increased the soluble chemical oxygen demand (COD) and accumulated VFAs in the effluent of the system (Meng et al., 2017). Here, cefalexin also increased the proportion of *Gelria* and *Syntrophorhabdus* bacteria and fungi, and the functional diversity of archaea.

The effects of cefazolin on methane production are variable (Beneragama et al., 2013; Alonso et al., 2019). On one hand, the addition of cefazolin reduced methane production by 39.8–68.3% during the anaerobic digestion of cow manure (Lateef et al., 2018). On the other hand, cefazolin did not inhibit methane production using the same digestion substrate and similar treatment duration (Beneragama et al., 2013). Effects are also variable for penicillin. For instance, Masse et al. (2000) showed that methane production of anaerobic digestion of pig feces is reduced by 35% due to the presence of penicillin at a concentration of 16 mg/kg in the feed. Whereas, penicillin was found to stabilize the anaerobic digestion process of rain tree kernels and thus to facilitate the methane production (Viswanath and Nand, 1989). Overall, the addition of β-lactams modifies microbial communities and induce variable effects on methane production.

#### Antibiotic Mixtures

Antibiotics are usually occurring as mixtures in waste and contaminated ecosystems, thus calling for the study of the effect of antibiotic mixtures on microbial processes. For instance, Ozbayram et al. (2015) tested the effects of sulfamethoxazole-tetracycline, erythromycin-sulfamethoxazole, and erythromycin-tetracycline on methane production. They found that methane production is inhibited in reactors fed with erythromycin-sulfamethoxazole and sulfamethoxazole-tetracycline, while the mixture of erythromycin-tetracycline showed only a weak inhibition. Inhibition on acetate utilization and methane production followed similar trend, which suggested the potential effect of antibiotic combinations on acetate digestion.

Inhibition of methane production with three antibiotics together, e.g., erythromycin-sulfamethoxazole-tetracycline, was higher than that with erythromycin-sulfamethoxazole; but lower than that of sulfamethoxazole-tetracycline, and erythromycin-tetracycline (Aydin et al., 2015a,b; Ozbayram et al., 2015). As a consequence, tetracycline may have a synergistic effect with erythromycin and sulfamethoxazole, respectively, while the presence of erythromycin with the other two antibiotics may produce an antagonistic effect. In the same investigations, similar trends were observed for the effect of antibiotic mixtures on acetate consumption, implying that inhibition of methanogenesis is due to a reduction of acetate consumption.

A mixture of oxytetracycline and chlortetracycline reduced methane production by more than half, indicating a much stronger inhibition effect than a single antibiotic (Alvarez et al., 2010). Here, authors proposed that the stability of oxytetracycline and chlortetracycline was favored by their strong adsorption to solid matter. Christensen et al. (2006) also observed a major synergic effect of erythromycin and terramycin on methane production in activated sludge samples. Furthermore, a study reveals that the inhibition effect of terramycin and cefazolin-terramycin mixture on methane production follows nearly same inhibition pattern during the thermophilic anaerobic digestion of cow manure (Beneragama et al., 2013). Overall, experiments on antibiotic mixtures show both synergetic and antagonistic effects, depending on the type of antibiotics.

#### Effects of Antibiotics on Methanogenic Archaea

Methanogens are a class of exclusive anaerobic archaea that are widely distributed and can survive in lakes, marshes, sludges, wetlands, rice paddy soils, inside organisms, and even extreme environments (Xiao et al., 2017, 2019b,c; Conrad, 2020). The blocking of the methanogenic reaction leads to the accumulation of organic acids, and the upstream acidification reaction will be slowed down by product inhibition.

Lamberti et al. (2011) suggested that bacteria and archaea have similar ribosome structures, because of which the macrolides and tetracyclines can inhibit methanogenic archaea. Roxithromycin made a positive contribution to the production of VFAs (Ni et al., 2020). The potential mechanism is presented as follows: acetate kinase activity was increased while the activities of alpha-glucosidase and coenzyme F-420 were decreased by the addition of roxithromycin; methane production was significantly inhibited than the hydrolysis process, which heavily accumulated VFAs content in the system. Moreover, inhibition of the acidogenesis and methanogenesis in waste activated sludge anaerobic digestion was observed for roxithromycin, resulting in decreased methane production. *Methanoseata*, *Methanofastidiosum*, and *Methanolinea* were key methanogenic archaea members in these systems but their proportion decreased in the presence of roxithromycin (Ni et al., 2020).

Interestingly, the impact of tylosin on methanogens and/or methanogenic progress seems to be slight compared to other macrolides antibiotics (Chelliapan et al., 2011, 2014). Chelliapan et al. (2011) showed an adaption of methanogens to tylosin, where archaeal cells were not inhibited by tylosin at the concentrations between 100 and 400 mg/L and dominated the reactor. Especially, methanogenesis was not inhibited in the digesters acclimated with 0.01 to 0.065 mg/L tylosin, and methane production was increased (Garcia-Sanchez et al., 2016). It could be inferred that not only the resistance but also the metabolizing ability to antibiotics were developed by such microorganisms. Tylosin effects on manure degradation were limited as well (Stone et al., 2009). Thus the consumption of acetate and other C-1 VFA compounds, such as formate, during methanogenesis was sufficient even with tylosin in the system.

Like the inhibition mechanism on bacteria, terramycin significantly inhibits acetoclastic methanogenic archaea at low concentration, and further enhances the inhibition ability at high concentration (Arikan et al., 2006). In contrast to terramycin, at a low concentration of aureomycin (25 mg/L), acetoclastic methanogens are not affected. However, at concentrations higher than 200 mg/L, the consumption of acetate is halted, and the whole methanogenic progress is inhibited (Sanz et al., 1996). Some studies showed that tylosin has no noticeable effect on acetoclastic methanogens, but only inhibits the oxidation of propionate and butyrate at high concentration. Unlike tylosin, erythromycin directly inhibits acetate-based methane metabolism (Sanz et al., 1996; Zhang and Li, 2018).

Cefalexin, one of the β-lactams, mainly affects methane production by inhibiting acetoclastic methanogens, thus accumulating VFAs (Lu et al., 2014). With the extension of cefalexin action duration, methanogenic archaea and anaerobic fermenting bacteria can adapt, and methane production tends to be restored. However, there is only a partial recovery in the methane production due to excessive cefalexin (Lu et al., 2014). The decline in the inhibitory effect of cefazolin in the later stage of the reaction might be due to the cefazolin which is easy to degrade under thermophilic environments (Fabre et al., 1994).

## Perspective and Conclusion

Antibiotic residues in sludge treatment systems often adversely affect the anaerobic digestion process, depending on conditions, antibiotic nature and antibiotic concentration. Research progress and knowledge in this field have been significantly enriched in the past ten years. Based on the current progress, some rules of antibiotics’ impacts on anaerobic digestion are summarized as follows:

(1) The presence of antibiotics generally lead to the accumulation of VFAs in the anaerobic fermentation system. However, different types of antibiotics or even different antibiotics of the same type have different influences on the anaerobic digestion process, the methane-production capacity, and the related microbial community.
(2) Besides the type of antibiotics, the concentrations, the duration of action, and the temperature of the anaerobic digestion system altogether considered to be the key factors affecting the effects of antibiotics on anaerobic digestion and methane production.
(3) The short-term and long-term effects of antibiotics show certain differences. Short-term experiments may be difficult to accurately reflect the potential effects of antibiotics on complex microbial consortia due to the lack of microbial adaptation. Short-term experiments ignore the long-term effects on the growth of anaerobic microbes and the adaptability of microbes to antibiotics. Thus, long-term observation and comprehensive analysis are to be given topmost priority. In the later stage of the anaerobic digestion reaction, some antibiotics lose their bacteriostatic effects. The possible reasons for such happenings are as follows: (1). the hydrolysis reaction of antibiotics is natural to occur; (2). bacteria and methanogens have developed tolerance to specific concentrations of antibiotics.

Antibiotics in sludge are generally not present alone, they are usually accompanied by other antibiotics. However, current research mainly focuses on a single antibiotic and its inhibition effect on anaerobic digestion. The lack of exploration and summary of mixed antibiotics’ impact on the anaerobic digestion system results in an incomprehensive knowledge on the joint effect of multiple antibiotics. Thus, it may not be instructive to practice under anaerobic conditions for wastewater and/or diverse antibiotic-enriched sludge treatment.

The effects of single or multiple antibiotics on anaerobic digestion of sludge depend not only on the antibiotics themselves but also on the sludge substrates, the microbial community composition, the biological and non-biological degradation of antibiotics, and the adsorption of antibiotics. These factors need to be further evaluated and will be the focus of future research. As environmental pollutants, ARGs require to be comprehensively studied in anaerobic fermentative environments, which is indispensable to characterize the impact of antibiotics. In contrast, the current techniques can only cultivate a small proportion of the anaerobic fermentative system members, which severely restrained our knowledge of the bacterial meta-resistome in various environments. Moreover, it wasn’t yet well documented the distribution of ARGs and antibiotic-producers and the effects of antibiotics on anaerobic digestion.

As a non-culture-based method, metagenomics becomes a vital tool to comprehend the bacterial communities sufficiently. Various omics techniques are more and more applied in understanding the effect of antibiotics on microbial communities, synthesizing new antimicrobial compounds, and analyzing the antibiotic resistance genes’ distribution in different anaerobic systems. For example, functional metagenomics can be applied in identifying novel antibiotic resistance genes, and descriptive metagenomics can be used for analyzing the composition of the microbial communities and catching the proportion of known antibiotic resistance genes (Garmendia et al., 2012; Gupta et al., 2020). Furthermore, metatranscriptome becomes other powerful analyzing tools for identifying ARGs and assessing the effects of antibiotics on the environment (Asante and Sekyere, 2019). Consequently, the application of omics methods will bring revolutionary improvements to the study of anaerobic digestion and methanogenic performance affected by antibiotics.

## Author Contributions

LX, YW, ZL, PK, JL, DF, QY, and FL collected the data. LX, EL, PK, and DF analyzed the data and wrote this manuscript. All authors contributed to the article and approved the submitted version.

## Conflict of Interest

The authors declare that the research was conducted in the absence of any commercial or financial relationships that could be construed as a potential conflict of interest.
